# Relational welfare: a socially just response to co-creating health and wellbeing for all

**DOI:** 10.1177/1403494820970815

**Published:** 2020-12-15

**Authors:** Dina Von Heimburg, Ottar Ness

**Affiliations:** 1 Nord University, Faculty of Social Sciences, Levanger, Norway; 2 Department of Education and Lifelong Learning, Norwegian University of Science and Technology, Trondheim, Norway

**Keywords:** Health promotion, co-creation, health equity, human rights, social justice, participatory parity, relational welfare, public value, sustainable development

## Abstract

**Aims:**

Contemporary approaches to pursuing public value and the vision of health and wellbeing for all have evolved notably in the past few decades, with distinct approaches termed ‘co-creation’ and ‘health promotion’ gaining traction. This article explores a critique of ongoing paradigmatic shifts in public health and the public sector, focusing on cross-fertilisation between co-creation and the promotion of health and wellbeing. Drawing on Nancy Fraser’s claims for social justice through redistribution, recognition and representation to achieve participatory parity, we discuss a need for transformative change to achieve societal goals of creating health and wellbeing for all, leaving no one behind.

**Conclusions:**

Health promotion and co-creation converge in a quest for active citizenship through participation, as well as embracing a whole-of-government and whole-of-society approach. However, inequity in such processes, as well as health and wellbeing outcomes, are still persistent and contradictory to health promotion aims. This article argues that radically attending to human relationships and our dependency on other humans as a ‘collective’ need to be placed at the core of future-forming social construction of public and democratic institutions to allow the ongoing cross-fertilisation between health promotion and co-creation to work. Responding to this calls for transformation, the article presents a framework for developing a relational approach to welfare. The framework advocates for ‘relational welfare’, which captures the intersection of the welfare state, democracy and human relationships attending to social justice, capabilities and health and wellbeing for all as key public values in societal development.

## Introduction

Health and wellbeing for all, leaving no one behind, are key public values for most governments, from global to local [1–3]. Health is a basic need and a human right, and equity in health and wellbeing are crucial for achieving sustainable societies [1,4]. Social inequities have toxic effects on societies. More unequal countries tend to be less healthy, have lower life expectancy and experience more crime and a range of other negative social outcomes [5,6]. However, persistent and widening inequities in health and wellbeing, within and between countries, remain an unsolved public health problem [2,6].

During the past few decades, parallel ideas, discourses and ideologies have been directed at tackling health inequities. Health promotion (HP) has been presented as a viable approach to widen the scope of public health beyond a biomedical approach to disease prevention, and explicitly links the value of equity to health and wellbeing as human rights [4,7–9]. Co-creation logic has recently gained traction in HP research, policy and practice [10], with co-creation presented as a viable path to tackle the complexities inherent in creating conditions for health, wellbeing and equitable outcomes [10–13]. Co-creation logic represents a participatory, collaborative, deliberative, multi-stakeholder and boundary-spanning approach to public value creation. This logic, aligned with a ‘new public governance’ approach, is rapidly becoming a core principle of reforming public sectors and democratic governance [14–17].

Co-creation is about ‘getting things done’ through collaboration [18], while HP aims to achieve health and wellbeing for all, leaving no one behind [2,4,7,9]. Although co-creation is suggested as a viable path to strengthen HP [10], research has shown that co-creation ideologies may in fact increase inequities in health and wellbeing [19]. Both Ostrom [20] and Steen et al. [21] argue that co-creation approaches fail to capture, and buffer, the effects on inequity. Moreover, HP fails to describe adequately equitable and socially just processes of successful co-creation [10]. The Nordic approach to welfare states is suggested as an ideal societal model in HP [22]. However, these states are also currently undergoing a transition, orienting towards co-creation [14]. Inequity in health and wellbeing remains a challenging problem, even within universal welfare states with generous redistribution of social transfers [2,22]. It remains a difficult task to translate small inequalities in wealth into small inequalities in health [23,24]. To discuss cross-fertilisation critically between HP and co-creation, even within HP ‘best practice societal models’, there is a need for an analytical framework to approach the political objectives of ‘health and wellbeing for all’ and ‘leaving no one behind’.

In times of transition and paradigm shifts affecting the public sector and welfare systems, we will argue that it is important to become aware of and empowered to tackle and buffer unintended and negative effects as well as identify and further potentials for achieving desired goals. In relation to this development, we will address the following potential paradox: the possibility that recent developments in HP and welfare creation can unintentionally increase social inequities in health and wellbeing, opposite to the societal goals of leaving no one behind. More specifically, we will discuss how possible unintended effects of recent developments in public health and the public sector can be understood and possibly addressed to pursue health and wellbeing for all.

Key concepts in co-creation and HP approaches are citizenship and community participation [4,7,17,25–27]. However, participation per se does not guarantee realising socially just outcomes in health and wellbeing. Thus, focusing on social justice in cross-fertilisation between co-creation approaches and HP aims in the public sector, we use Nancy Fraser’s theory of justice as a theoretical lens to guide a critical investigation of the presented approaches. Her conceptualisation of participatory parity will be used to explore how HP and welfare creation in the intersection of the state, the people and the wider society can be made socially just and beneficial for achieving health and wellbeing for all. As a conceptual basis for discussing these concepts, we will start by outlining the conceptualisations of ‘health promotion’ and ‘co-creation’.

## Health promotion

The World Health Organization (WHO) Ottawa Charter for Health Promotion defines HP as ‘the process of enabling people to increase control over, and improve their health’ [7]. This definition still dominates the HP field. However, the concept of ‘process’ is increasingly linked to a socioecologically oriented whole-systems approach to handling the complexity inherent in the social determinants of health [12,28–31]. HP can be described as a conglomerate of theoretical and practical approaches to achieve health, wellbeing and equitable outcomes as focal points for public value creation [32]. HP is generally conceived as an area for action rather than as a separate discipline [33] and moves the practice of public health from a biomedical, risk-oriented discourse towards the settings of everyday life, linked to human dignity and human rights [7–9]. A crucial acknowledgement within HP is the importance of the social determinants of health and wellbeing [34]. The social determinants connect to the conditions in which people are born, grow, live, work and age and to how creation of such conditions is shaped by the distribution of money, power and resources (i.e. social support and connectedness, education, income, housing, food security, employment, quality working conditions and participation in society and democracy) [2,6]. This is a shift away from sectorisation of governments in which different sectors make bids on their own appropriations. Instead, the focus is on pursuing equity in health and wellbeing as a common purpose, mobilising the whole of society and the whole of government across sectors and stakeholders at multiple levels [32].

The Ottawa Charter emphasises ‘creating supportive environments’ and ‘strengthening community action’ as core pillars of HP [7]. This ‘settings focus’ was further refined through the Shanghai Charter, which stated that: ‘health is created in the settings of everyday life – in the neighbourhoods and communities where people live, love, work, shop and play’ [4]. The focus revolves around the settings of everyday life in local communities, highlighting healthy and resilient cities and communities at the local governance level [4,26]. Further, HP is built on a salutogenic orientation [35,36], which focuses on assets to promote health and wellbeing rather than the aetiology of disease [37]. This is based on a conception of health as a continuum, in which health and wellbeing can also be achieved when people have a medical condition [8,35]. HP’s embrace of a social or human rights model of health and wellbeing to complement the biomedical model has strategic advantages as well as empirical support [38]. The focus is to facilitate actions on the many social, political and economic factors that create grave disparities for people and to support equitable outcomes [8,9,39]. More recently, aligned with theories of justice, assets for health and wellbeing are framed in HP literature through the lens of capabilities [6,34]. Sen [40–42] links the development of capabilities to freedom and quality of life. The focus is on liberating people to achieve the beings and doings (i.e. functioning) that constitute one’s wellbeing. This means accumulating capabilities that enable people to have a life they (themselves) have reason to value. However, assets and capabilities for health and wellbeing are highly influenced by social and economic arrangements, community resources and welfare state institutions [34].

According to the WHO [2], key drivers of health and wellbeing equity are empowerment, participation, coherent policies and accountability. These drivers relate to the following important principles of HP: a whole-of-government and whole-of-society approach, strong and invigorated governance and leadership for health and wellbeing across sectors and levels of government, collaborative models of working and shared priorities aligned with a radical focus on community and individual empowerment and resilience [2,30,43]. The Shanghai Charter [4] firmly connects HP to the UN’s Sustainable Development Goals (SDGs) by stating: ‘Healthy lives and increased wellbeing for people at all ages can be only achieved by promoting health through all the SDGs and by engaging the whole of society in the health development process.’ This statement is a demand for co-creation that places health, wellbeing and social justice at the heart of public value creation, interlinked with the ecology of sustainable societal development.

Contrasting HP with conventional public health can be intellectualised as an artefact. In practice, they are complementary approaches. However, they are constitutive for distinct discursive communities where HP is regarded as a paradigm shift and a game changer in public health [32]. As proposed by Kickbush and Gleicher [44], HP relies on a thorough participatory and collaborative imperative, which resonates with core principles in the co-creation approach. Based on the above-mentioned literature, we have developed core characteristics to contrast HP to conventional public health (see Table I). This distinction is constructed to outline a fundament for advancing a discussion on the cross-fertilisation between HP and co-creation in pursuit of health and wellbeing for all.

**Table I.** Core characteristics of conventional public health and health promotion.

Dimensions	Conventional public health	Health promotion
**Objectives**	Prevent the burden of disease	Promote health and wellbeing for all, leaving no one behind
**Focus and approach**	Individual, biological and environmental determinants of disease and disasters. Individual, linear and reductionist approach, focused on direct measures such as screening, targeted prevention and medical treatment provided by experts	Assets, resources and capabilities for health and wellbeing. Contextual, systemic and processual approach, focused on coordinated and integrated measures to mobilise appropriate actions in the settings of everyday life
**View of people**	Potentially ill, focus on individuals and reductionism	Active, participating, meaning-making subjects, focus on community and social ecology
**Actions**	What reduces risk of death and specific diseases based on diagnostic criteria	What empowers people and makes life meaningful and worth living
**Responsibility**	Health sector, health professionals, and individuals	Whole-of-government, whole-of-society, and political leaders

## Co-creation in the public sector

In general, the overall aim of the public sector is to create public value, just as the private sector focuses on the creation of private value [45,46]. After a long époque of classic bureaucratic rules, followed by a shorter spell of marketisation and performance management dominance, discussion about public value has increased in the public sector [17,18]. The rise of the discussion of public value and how such value can be created can be attributed to the seminal text by Moore (46) on the need to correct the neoliberal adoption of ‘new public management’ (NPM) in the public sector. Co-creation approaches, linked to a ‘new public governance’ perspective, have been developed as a critical response to classic bureaucratic administration and NPM [47,48].

There is an ongoing revolt against conventional forms of policy-making and public sector services, advocating for exploring more democratic and participatory involvement that not only generates sustainable policy change but also empowers community-oriented practices [49,50]. Reasons for such developments are driven by the complexities of contemporary societal problems, such as sustainable development and persistent inequities in health. In developed welfare states, another important driver is that the public sector seems to be ‘caught’ in a crossfire between growing public expectations and severe fiscal constraints [17,51]. This calls for innovation in the public sector [17,47,52]. Co-creation is presented as a viable response, aiming to transform the conceptualisation of the public sector from an authority and a service provider into an arena for co-creation [51].

The co-creation narrative evolved during the past decade at an accelerating pace. The aim has been to redefine central logics in the public sector and democracy [17,51]. The basic idea in co-creation is to allow and empower citizens to become actively involved in creating public value, in which governments aim to involve citizens and organisations dynamically in participatory processes to solve social and political problems. Co-creation is suggested to promote a variety of desirable outcomes, such as increasing citizenship, deepening democracy, developing user-centred and high-quality public services, increasing efficiency, bolstering innovation and facilitating community mobilisation [17,18,48,49,51–54]. These developments have led ‘co-creation’ rapidly to become a buzzword in the public sector [14,15], widely embraced by public managers, political scientists and public administration researchers. We will not go into detail on the multitude of concepts and terms related to co-creation logic; rather, we will build on the arguments for adopting co-creation as an umbrella term in this paradigmatic shift, encompassing co-production and other related concepts [15,18,55]. Here, the term co-production often refers to a dyadic relationship of actors engaging in voluntary or involuntary production in any public service (i.e. between a service user and a public servant). As argued by Osborne et al. [56], one cannot have (public) service delivery without co-production. Encompassing co-production, co-creation often entails the whole policy process, including a political dimension [17,25]. Torfing et al. [51] provide a general definition of co-creation in the public sector:

a process through which two or more public and private actors attempt to solve a shared problem, challenge, or task through a constructive exchange of different kinds of knowledge, resources, competences, and ideas that enhance the production of public value in terms of visions, plans, policies, strategies, regulatory frameworks, or services, either through a continuous improvement of outputs or outcomes or through innovative step-changes that transform the understanding of the problem or task at hand and lead to new ways of solving it.

Although different approaches to co-creation co-exist, they generally have in common that they change the role of citizens from passive (disempowered/obedient receiver and demanding consumer) to active, empowered contributors in the process of (co-)creating public value. Relevant and affected actors are presumed to participate in defining and solving shared problems and common tasks [51]. This entails recognising and accumulating resources and capabilities in and between people, places and communities. In the co-creation literature, public value creation is fundamentally linked to the ‘wider life experience’ of citizens and service users, in which public value is created in the nexus of interactions between stakeholders, at different levels [55]. Accordingly, within the co-creation perspective, public service users (i.e. citizens) tend to be positioned as the main value producer rather than the public sector organisation. Moreover, co-creation of public value can happen at all stages in the policy process, including co-initiation, co-design, co-implementation, co-production and co-evaluation [17,25,51].

The co-creation approach implies transformative ways to achieve welfare. Local-level governments are then primarily conceptualised as a place and a local community to foreground an organisational ‘public service’ discourse. The guiding principle is that the local community is best developed through a joint focus on tasks, opportunities and problems [18,51]. This means that the main task of the municipality is understood as mobilising people’s assets, supporting them to cope with everyday life, strengthening social networks, increasing community participation and empowering local communities. The focus is directed towards what citizens, volunteers, businesses, professionals, leaders and politicians can achieve through joint action and collaboration, both at the interpersonal level and across organisational boundaries and levels of government [15,25,51].

Defining the spirit of co-creation could be described as a logic in which the creation of public value (including welfare) is achieved by collaborative processes in which the public sector creates value with the citizens [51,55]. New ideas on co-creation in the public sector do not fully replace existing ideas and practices but rather co-exist with ideas entrenched in classic public bureaucracy and NPM (conventional approaches) [16]. Although the co-creation approach adds to existing logics in the public sector and democracy, it simultaneously tends to clash and create tensions with institutionalised government norms and practices. This development of the co-creation logic is closely connected to ideas and principles that have shaped HP since the mid-1980s [10]. In the same way HP represents a ‘paradigm shift’ in public health, co-creation is presented as an equivalent radical change of approach in the public sector. Based on the above-mentioned literature on co-creation, we have developed conceptual distinctions between conventional public administration and management and co-creational approaches to public governance (Table II) to provide a conceptual fundament for our further discussion.

**Table II.** Distinctions between conventional public administration and management and co-creational approaches to public governance.

Dimensions	Conventional public administration and management	Co-creation in the public sector
**Objectives**	Public sector authority, economic efficiency and service satisfaction	‘Getting things done’ to create public value
**Focus and approach**	Public sector organisations are approached as separate entities. Creation of quasi-markets to enhance competition. Hierarchical steering and horizontal division of labour	Creating public value through collaboration (whole-of-government, whole-of-society, multi-level). Cross-boundary and multi-level collaboration among relevant and affected actors from the public, private and civic sectors
**View of people**	Passive clients or demanding consumers. Focus on individuals and their rights and needs	Active contributors to co-creating public value outcomes. Focus on networks and their capacities
**Actions**	Segregated actions between sectors and professions	Empowerment of stakeholders beyond organisational boundaries, seeking to mobilise community action
**Responsibilities**	Elected politicians exercise sovereign leadership, where public managers are expected to ensure a rule-bound administration controlled by performance management. Citizens play a marginal role between elections	Public leadership is horizontal, distributive and integrative. Elected politicians exercise leadership to mobilise their communities and pursue ongoing democratic deliberation and joint accountability

### Socially just co-creation: redistribution, recognition and representation as prerequisites

An attempt to advance the discussion on creating approaches and conditions to achieve HP aims should include the role, function and further development of the public sector, how it is governed and how participation and governance connect to democracy [2,27]. The HP agenda and co-creation are equally based on premises of active citizen participation, empowerment and community mobilisation. However, they have contrasting focuses. As mentioned earlier, co-creation is about ‘getting things done’ through collaboration and crowd-sourcing of resources [18], while collaborative processes in HP specifically aim to achieve health and wellbeing for all, leaving no one behind [4,7,9]. If collaboration is imperative in HP [44], and social justice is placed at the heart of reaching the goals of creating health and wellbeing for all [2,30], central questions to be addressed regarding cross-fertilisation between co-creation and HP should be: Who should participate? Who invites and who decides? How? Where? And with what purpose and consequence? These questions concern how participation can lead to fairness, how the public sector should be governed, and how to approach much-needed innovation to develop sustainable welfare solutions. We will argue that these are not only matters of theoretical consideration but also questions about normative and transformative social change for sustainable development and social justice aligned with human rights. Critical social theories suggest that power works to shape society in ways that are self-reinforcing for the powerful but changeable through critical reflection attempting to understand how society works to do so [57]. Thus, we claim that it is crucial to understand what factors and decisions are driving inequities and voice the concerns of those who are deprived of opportunities for a good quality of life. If not, the focus could remain on describing the problem, not on identifying solutions and striving towards socially just outcomes.

We have found the ‘theory of justice’ of Fraser [58,59] to provide a useful theoretical lens to critically analyse the cross-fertilisation between co-creation and the pursuit of health and wellbeing for all. Fraser [59,60] views social justice as a social arrangement that makes it possible for all human beings in a society to participate on equal terms in social life. She calls this ‘participatory parity’ (i.e. how humans are able to participate as equals, when ‘participation’ is the main purpose and desired outcome). Her theory of justice has a transformative purpose in which participation on equal terms serves as a normative reference point. She not only criticises injustice and focuses on the moral good of participation in social life on equal grounds but also explains how participatory parity is anchored in the ways societies function and how justice can be implemented [59]. At first, Fraser [61] saw participatory parity along two intertwined axes: ‘redistribution’ (i.e. economic justice) and ‘recognition’ (i.e. cultural justice). Later, she included the concept of ‘representation’, arguing that justice acquires a political dimension [60]. Together, these three dimensions encompass vital components of transformational social change in a just direction. According to Fraser [59,60], maldistribution, misrecognition and misrepresentation are unfair for the same reason: they involve a breach of the norm of participation on equal terms.

Fraser [62] claims that change, to meet everyone’s needs and ensure social justice, can only be achieved through radical societal transformation. To explore the transformations proposed by HP and co-creation, a useful and parallel argument is presented by Fraser herself. She critically discusses second-wave feminism through what she calls a ‘disturbing possibility’, pointing to unintended negative effects [59]. She argues that the cultural changes that were jump-started by second-wave activists served to ‘legitimate a structural transformation of capitalist society that runs directly counter to feminist visions of a just society’ [59]. A similar critique is seen with respect to the embrace of co-creation perspectives in the public sector – the disturbing possibility that the co-creation logic, despite the approach’s participatory ambitions, could legitimise measures and politics that in fact may increase inequity in health and living conditions. Fraser [59] emphasises three dimensions in her theory of justice: redistribution, recognition and representation, providing conceptual guidance to our further discussion.

### Redistribution

Redistribution means the fair allocation of divisible goods, with these goods typically economic in nature and relates to the class structure of society. Such goods are associated with a wide range of resources (i.e. wealth and access to welfare) that are necessary to interact with others as peers [59]. Redistribution is a central aspect to achieve equity in health [30,39]. However, this aspect might be threatened by adopting a ‘co-creation logic’. First, co-creation could support a legitimation of policies that make health and wellbeing (only) an individual responsibility, which can lead to displacing the responsibility of the state in pursuing health equity as a public value. If the notion of ‘shared responsibility’ between citizens and the public sector is (dis)placed in a neoliberal, market-oriented and individualistic rationale, the co-creation logic (i.e. mobilising resources and capabilities) could lead to a legitimation of a ‘blaming the victim approach’ [63]. Fraser [63] argues that recognition, if left alone in a one-dimensional approach to justice, could contribute to an individualistic ‘psychologisation’ of common concerns; that is, characterise the struggle for recognition as a question of individual or interpersonal psychology. Such an individualised approach to participation in co-creation may risk failing to identify and tackle the ‘causes of the causes’ of health and wellbeing, which are deeply embedded in redistributive strategies (i.e. redistribution of determinants of health, such as income, housing and welfare services) [6].

Second, although the asset perspective and the ‘relational turn’ in co-creating welfare is initially promising, solutions that are distributed but individualised may increase the social gradient in health and wellbeing [19]. This is because they can depend on people’s access to resources, which also affects their ability to access them. Citizens with high socioeconomic status tend to participate more in co-creational processes than fellow citizens further down the socioeconomic ladder. More privileged citizens tend to have assets (e.g. power through social networks, money, education and work positions) that accumulate participation [20,21]. If not properly addressed, unequal access to such assets could widen the health gap.Third, endorsing a rationality in which multiple actors and organizations are welcome to participate might produce a dark side of outsourcing welfare. Torfing et al. [51] claim that co-creation is built on ‘collaboration with relevant and affected actors who can help to define and solve the shared problems and common tasks’. We will argue that ‘collaborative imperatives’ within the co-creation logic could (unintentionally) legitimise political arguments for the privatisation of welfare, pursuing the interest of economic capital on behalf of human and social capital. Our concern is that this problem could grow as corporate interests tend to outweigh the voices and presence of marginalised groups in policy processes. Furthermore, the ‘imperative’ of citizen participation can run the risk of disguising or unintentionally contributing to dismantling the welfare state and all public sector organisations bringing it into action, legitimising an argument for welfare state retrenchment. If the threshold of public sector responsibility is lowered, lack of accountability could threaten the distributive effect of welfare systems as capabilities and resources are unequally distributed in and among societies, local communities, groups and individuals.

### Recognition

Recognition is essential to the development of a sense of self and to being acknowledged, loved and valued as an equal peer and for one’s specific identities, such as gender, race, age and sexuality [59,61]. Love, recognition and the necessity of belonging with others are basic human needs [64] and are recognised as fundamental components in the social determinants of health and wellbeing [34,65]. Research on co-creation demonstrates that social capital, the ties that bind communities together, is vital for successful co-creation to achieve desired outcomes. This is especially linked to relationships built on trust and reciprocal sharing of power [18]. When using co-creation in HP and linked to the health equity agenda, we suggest that the following questions should be addressed: Who is recognised? By whom? Where, what, when and how? If cultures of recognition do not include the whole of populations, we fear a widening gap between ‘us’ and ‘them’ instead of a return to an inclusive and diversified view on merit.

Contemporary societies are becoming more segregated and polarised, and levels of social trust are declining [66]. If people across different cultural and socioeconomic groups do not meet and greet each other and try to overcome barriers of unbalanced power relations through dialogue, this might create a divide concerning who is granted merit in co-creational processes [21]. We emphasise that if such segregation is not properly addressed, it can contribute to negative discrimination in who provides value in the co-creation of ‘public value’, resulting in dis-recognising and dis-engaging those in the lower portion of the social gradient and thus increasing the health gap.

### Representation

Representation, according to Fraser [59], furnishes the political stage on which struggles for redistribution and recognition play out. It points to social belonging in decision-making processes (the ‘who’) and the procedures that structure democratic processes (the ‘how’). Democratic participation is a major issue in the co-creation literature arguing for deepening democracy through enhancing participatory and inclusive citizenship and reviving democratic values through deliberative democratic models [17,51]. Public deliberation is based on reasoned argumentation between free and equal citizens who are brought together to talk about collective ideals, problems and possible actions that might advance a public interest [67]. However, some major concerns need to be considered. Following Fraser [60], we are concerned that by disputing the ‘what’ of justice, participants may neglect the necessity to dispute the ‘who’. Furthermore, practices of ‘closed circles’ of political power threaten the voices of those outside these circles. If democratic deliberation is not carefully designed and facilitated, it could unintentionally increase polarisation, generate frustration and reinforce democratic echo chambers that create adverse effects in creating wellbeing opportunities [67,68].

Citizens at the lower end of the social gradient may act as ‘blind spots’ in the eyes of more privileged citizens [39,69,70]. This is because most people further up the social gradient do not directly face struggles of deprivation and discrimination in their everyday lives. Disadvantaged citizens may be constrained in their contribution to co-creation by a lack of knowledge (on how to co-create and the importance of their input) and by a lack of material conditions (that promote their input) [19]. Feelings of shame and lack of confidence to participate can also be a barrier [71]; not being present, or feeling alienated, excluded or neglected by the dominant discourse, may affect possibilities for voicing and representation [27,67,70,72]. A consequence can be that democratic processes privilege participation by groups high up on the socioeconomic ladder and fail to empower and advance the interests of those less privileged or misrecognised by cultural struggles.

## Relationships as the missing link in achieving health and wellbeing for all?

If health and wellbeing equity is placed at the heart of public value creation, questions are also raised as to what this involves in practice. Public health problems such as unemployment, loneliness, bullying and lack of social support cannot be solved solely by available measures in the public sector. Humans are relational beings and always depend on each other to survive and thrive in communal forms of organising human collectives [73–76]. Social support and social capital are some of the most vital assets for health and wellbeing, but these values cannot merely be ‘delivered’ as a public service [65,77]. Recognising the social construction of public institutions and the importance of relationships calls for transformative change; how citizens’ and stakeholders’ participation impacts the creation of health and wellbeing is at stake.

We propose deepening the discussion with a simple yet complex response: Transformative change is bound by human relationships, and these relationships are the core of the fluid and dynamic process of socially just participation and public value creation.

Loving and supporting human relationships are essential for human health and wellbeing. A significant body of research suggests that such relationships are the most vital of all social determinants for achieving health and wellbeing [49,65,66,78]. In addition, the quality of relationships between public service users and public servants (i.e. in therapy or in kindergarten or other school settings) tends to be the most important factor for successful outcomes (i.e. learning, wellbeing) [79–81]. In addition, the importance of attending to relationships is increasingly acknowledged as vital for organisational functioning and interactive forms of governance and public service delivery aligned with co-creation [82,83]. Opposite to these facts, neoliberal societal tendencies that escalate individualism, competition, expert dependency and social divides still contribute to alienate humans as relational beings and ignore our dependency on the ‘collective’ [74,75]. Thus, in addition to Fraser’s three Rs of redistribution, recognition and representation to achieve participatory parity, we therefore propose a fourth R: relationships. Human relationships, in which people attend to communal forms of interaction, merit substantial attention if equity in health and wellbeing is to be achieved.

### A relational account of public value

According to Meynhart [84], public value is created when organisations fulfil basic human needs. Meynhart [84] adopts a subjective, psychological and emotional-motivational account of public value, arguing for a grounding in individuals’ representations and interpretations, stating that ‘public value starts and ends within the individual’. Radical attention to relationships challenges a view of public value as an aggregated sum of individual values. Rather, a relational epistemology perceives public value as constructed through interactive meaning-making processes that are highly dependent on human relationships, social interaction and wider context [74]. Constructing conceptions of values as well as social roles with actors in society in ways that are socially just is bound up within a broader ecology affecting meaning-making processes and processes of relating to others [73]. Role perceptions and self-understanding formed by relationships create symbols, interactions and ways of being in social worlds that impact people, places and the planet [73,85].

We support the original approach of Moore [46] to public value as a normative account that is related to social justice. The agenda of creating health and wellbeing for all, leaving no one behind, has recently been framed through a lens of supporting capabilities as a moral and human rights imperative but also as a prerequisite for sustainable development [6,34]. Sen [40,42] makes a compelling argument for replacing economic imperatives in societal development with the freedom to achieve wellbeing. He argues that policies and practices should concentrate on people’s quality of life, the conditions affecting possibilities to live lives people have reason to value and what people are able to do and be through the accumulation of capabilities. Nussbaum [86] argues that nation-states are morally bound to ensure that central capabilities are provided to their citizens. In other words, within a ‘public value discourse’, the concept of capabilities is placed at the core of public value creation [40,86,87].

According to Dréze and Sen [88], developments of capabilities should not be mistaken for individual processes. Social opportunities are described as a crucial prerequisite and a reminder not to view individuals and their opportunities in isolated terms. Sen [40,42] makes an argument for not predefining a list of capabilities but rather for a deliberative approach to prioritising core capabilities to pursue, arguing that nurturing capabilities must be sensitive to geographical region, social history and cultural values. Both Sen [40,42] and Pūras [9] support a normative approach to socially just public value creation but one in which processes of pursuing capabilities are negotiated and situated between actors, relating to each other, in specific contexts.

Building on McNamee and Gergen [89], we suggest that generative and socially just participation aligned with relational coordination between citizens and stakeholders should be made a focal point in the pursuit of creating capabilities leading to health and wellbeing for all. In such a perspective, the focus is on relations and social worlds to foreground services and professionals and cooperative networks to accomplish joint tasks to foreground sectorial division of labour [77]. A transformative focus on relationships, and dialogues inherent in such, places special emphasis on community belongingness and the relational responsibility of society as a collective [89]. Incorporating a relational dimension thus makes an argument for replacing a neoliberal individualistic responsibility with a focus on relational responsibility, radically attending to the process of relating and deliberating through transformative dialogue and joint action [89]. We will argue that radical attention to relationships could support a societal movement towards health and wellbeing for all through a relational recovery of society [65,74,77].

## Relational welfare: a viable response to socially just co-creation of public value?

Recently, the notion of ‘relational welfare’ of Cottam [77] has gained traction, pointing to the need to revolutionise the relationship between people (i.e. the public) and the welfare state (i.e. the public sector). Relational welfare is described as a ‘radical change’ in which relational bonds between human beings should serve as a starting point for promoting health and wellbeing in the 21st century [77]. Here, the concept of welfare connects to the very objective of welfare: to live well and flourish and nurture capabilities for doing so.

If co-creation is about ‘getting things done’ and increasing efficiency, and societies aim to achieve health and wellbeing for all, an important question to be addressed through a relational welfare perspective is: How can such a radical change be relationally ethical and socially just? We are aware that it is impossible to provide a complete answer to this question. However, if welfare is essentially co-created [56], in which health and wellbeing for all as public values are created in the nexus between actors in complex, adaptive systems [10,29] and lenses then contour and colour how we see such ‘systems’ must also be revisited in a reimagination of welfare creation. Empirical research and theoretical guidance can provide some insight to formulate common themes that combine co-creation, HP and social justice, suggesting that health and wellbeing for all might be achieved through advancing relational welfare. Such an approach could advance the fluid and complex relationship among the welfare state, the settings of everyday life and community development attending to relationships and participatory parity.

To mobilise and promote the conditions for revitalising the social links and trust that enable societies to thrive, the concept of ‘community’ is vital [76,90,91]. However, importantly, the further discussion is not about alternative models of welfare state retraction, such as the co-creation legitimised as ‘Big Society’ reform in the UK, which basically led to widening health inequities there [92]. Turning to evidence on best-practice societal models to achieve health equity, we propose an advancement of relational welfare that involves protecting and promoting a strong public sector that safeguards the principle of redistribution within a universal, inclusive and generous welfare state as suggested by Raphael [22].

By understanding health and wellbeing as public values created in all parts of society, we believe that it makes sense to see public services as an integral part of promoting health and wellbeing in people’s everyday lives. For example, public institutions (e.g. kindergartens, schools, libraries, nursing homes) could not only function as places where people receive welfare and public services but also serve as community centres and meeting places in the setting of everyday life, with the potential to create bonds and bridge gaps among people, groups and organisations and policy processes [28]. Accordingly, many actors can contribute to the creation of capabilities, in many ways, in many arenas. Achieving healthy lives and wellbeing for all requires action across multiple sectors, arenas and domains and commitment and know-how from a range of workforces and stakeholders outside of the health sector [6]. This entails enhancing capacity building to co-design and facilitate processes and take on boundary-spanning roles to empower joint action among citizens, stakeholders and sectors. Such a shift is a movement away from transactional logic and towards a collaborative model in which welfare is not purely delivered as a service but rather is brought to life by being created together [77,83].

A relational turn to welfare, building on co-creation and HP, redefines the role of the citizens, which means building on what is strong rather than what is wrong. This means seeing people and communities as capable citizens, not as defective clients [50,77,91]. A capability-oriented approach supports an acknowledgement that citizens have a valued social role through citizenship [93], in which all humans, to varying degrees, alternate between being people who need help and being people who might provide support. Advancing relational welfare should recognise inclusive participation in every aspect [77]. This also involves respecting people’s right not to participate [94]. The challenge will be to identify, mobilise and connect resources in local communities to create joint action in socioecological systems that provide the opportunity to receive and provide support as needed [2,6,50]. Importantly, we recognise that viewing local governments as arenas for creating relational welfare should not detract from the public sector’s responsibility and accountability for public welfare aimed at achieving health and wellbeing for all. On the contrary, this means that public services should be integrated into the local community, squarely placing accountability on (political) leaders in all parts of society.

Relational welfare through participatory and co-creational approaches has the potential to be transformative and health promoting if these approaches are carefully supported. This entails redressing power imbalances where possible and at the same time acknowledging, addressing and being transparent about the remaining imbalances [94]. Such a change requires a transformation towards more inclusive and relational organisational cultures and structures, where public sector organisations acknowledge that their actors and ways of working must change instead of (dis)placing responsibility for change on the citizens [94]. If principles of justice and inclusion are to be maintained, we agree with Fung [95] and Hanefeld et al. [68] that co-creational approaches to democratic governance (participatory and deliberative approaches) can not only inform and deepen democracy but also accelerate fair societies. Democratic processes should ensure that the different needs and perspectives of groups at risk of marginalisation are actively involved and recognised in policy processes, with effective measures for inclusive participation [70,72].

Democratic innovations based on socially just co-creation can connect public services and welfare institutions as democratic arenas. Universal approaches to welfare have the potential to bring places and spaces in line with the democratic call for processes of Lister [94] that are open and conducive to broad and inclusive democratic participation, in which politically marginalised groups are recognised and supported as subjects in policy-making. Universal welfare institutions, such as kindergartens, schools and libraries, could enhance participatory parity by linking participation in one arena to other arenas and policy-making processes.

As argued earlier, issues of health equity need to address the wider social determinants of health. For the purpose of achieving equity in health and wellbeing, we follow the argument for upholding the redistributive effects of Fraser [61,63] as a fundamental principle to achieve justice. However, political will for redistribution aligned with universal and generous welfare systems is formed and informed by citizens. To what extent citizens who live in marginalised and vulnerable living situations are invited, welcomed and recognised in democracy and policy processes thus affects policy development and policy outcomes [2,27,96–98]. Protecting and promoting societal goals of health and wellbeing for all thus starts with people, shoulder to shoulder, relationally attending each other as a collective in which participation is made easy, intuitive and natural [77,98]. Based on the arguments we have presented in this article, we urge infusing the political discourse with curiosity instead of polarised debate and kindness and empathy instead of hateful rhetoric. Basically, this is a matter of relational responsibility [89]. However, such relational responsibility needs to be accompanied by relational accountability, with equity in health and wellbeing, supported by creating capabilities as core societal objectives. Thus, the arguments outlined support the normative approach to public value of Moore [46]. Politicians, public sector leaders and public sector organisations should therefore systematically be held accountable for the population’s quality of life and for levelling up the social gradient [2,30].

### A typology of relational welfare

Relational welfare makes people and the relationships between them a focal point to reinvent and design societies and welfare systems, placing accountability squarely on the public sector and political leaders. Ness and von Heimburg [99] recently proposed a definition of relational welfare:

Relational welfare is a human centred and collaborative approach premised on human rights, social justice and societal sustainable development. Relational welfare means that welfare is a resource that people co-create together, where personal and collective relationships and environments are placed at the centre of development. Within this, foremost mission of the public sector is to build public value as a common good by supporting conditions that enables all people to flourish and live a life they have reason to value, and the capacity to sustain. The purpose is to strengthen the resources, relationships and communities to create positive and sustainable life courses, now and in the future.

To summarise the propositions outlined in this article to advance relational welfare that adopts a grammar of justice, we propose a conceptual framework describing key and interdependent concepts to guide a socially just and relational creation of welfare (see Table III).

According to Pūras [9], there are several threats to human rights and opportunities to come together in solidarity to rethink and reshape social, economic and political structures to ensure a sustainable, peaceful, just and inclusive future. These threats relate to authoritarianism, late-stage neoliberalism, climate change, paternalism and the rise of big data. To pursue transformative change, Pūras argues for governance systems that ensure responsibility and accountability for securing health and wellbeing as human rights and universal public values. Such a rights-based approach to the highest attainable standard of health is built on human rights as well as committing to the SDGs and makes a normative argument for health and wellbeing as public values [8,9,100]. We suggest that the ‘world-first wellbeing budget’ adopted by New Zealand in 2019, and up-scaled by the Wellbeing governments network (WEGo), is a practice of government that mirrors public value as a common good. By prioritising health, wellbeing, social justice and sustainable development over gross domestic product, this practice merits upscaling to other contexts and levels of government [9,99,101].

We propose that if the logic of co-creation is cross-fertilised with HP within a solid, distributive and multi-levelled welfare state, this might serve as a promising avenue to promote coherent and integrated actions that are imperative to create fair societies and healthy lives for all. All levels of government should continuously be held accountable for inclusive participation in the co-creation of public value as well as policy outcomes attending to health and wellbeing for all, leaving no one behind [2,30,102]. We propose that developing a typology for developing the ideas and practices of ‘relational welfare’ attending to redistribution, recognition, representation and relationships could support such a quest. Table III outlines a framework for advancing a typology of relational welfare.

**Table III.** The 4 Rs of relational welfare.

**Redistribution**	• Address the root causes of social inequities • Ensure equal access to universal welfare within a generous and distributive welfare state attending to the places where people live their everyday lives Support citizens to accumulate capabilities. Support is proportionally distributed and prioritised according to needs (individuals, groups, places and settings)
**Recognition**	• Ensure inclusive citizenship through recognition of diversity • Citizens are universally recognised and appreciated for who they are • Create trust, possibilities and empowerment through a people-centred, family-oriented and community-based approach Recognise, identify, connect and mobilise resources and capabilities in people, settings and places
**Representation**	• Support as key public value capabilities for all that are governed by the people, with the people, for present and future generations (attending to sustainable development) • Democratic institutions are inclusive in terms of presence and voice • Citizens who are deprived of opportunities to develop capabilities are actively and proportionately represented in democratic dialogue on issues affecting their lives • Elected politicians take on leadership towards health and wellbeing for all and engage in reflexive deliberation with citizens and stakeholders • Politicians and public sector employees across sectors and societal levels facilitate empowerment to ensure inclusive participation Politicians are held responsible for health, wellbeing and equity as a common good by transparent accountability systems that integrate public sector governance in democratic processes and political leadership
**Relationships**	• Act as the starting point, driving force and outcome of transformative processes that make participation easy, intuitive and natural • Support shared visions of justice through relational meaning–making processes • Nurtured by acts of empathy, kindness and relational responsibility in community life, public services and policy processes • Facilitate joint action through coordinated, boundary-spanning and collaborative networks (relational coordination) Enhance a valued social role for all citizens through processes of relating to others and participating in community life, taking actions to accumulate capabilities with others in their community and beyond

## Closing reflections

Marmot and colleagues [6,39] place equity in health and wellbeing at the very heart of public value creation when they conclude that fair societies nurture healthy lives for all. Increased citizen and stakeholder participation can be associated with transformative aims, as more inclusive and participatory methods are often associated with better wellbeing and health outcomes. Marmot and colleagues [39] state that: ‘without citizen participation and community engagement fostered by public service organisations, it will be difficult to improve penetration of interventions and to impact on health inequalities’. However, explored in this article, participation per se does not make such processes or policy outcomes socially just [72,96]. Cross-fertilisation between co-creation with HP aims must therefore focus on social justice and attend to human relationships in every aspect. The process of relating and participating is systemic and reciprocal. A focus on participatory parity, relational responsibility and equal opportunities to develop capabilities could advance a just ordering of social relationships within a society and shift the grammar of welfare to a grammar of democratic justice, as suggested by Fraser [59]. Such a grammar could be advanced by developing a relational approach to welfare, adopting principles from co-creation and HP, and placing accountability for outcomes in the public sector and political leaders. We suggest that the presented framework for developing relational welfare could serve as a viable response to the recent call of Lundberg [103] for reframing and rethinking common beliefs and practices to tackle the social determinants of health.

Based on our theoretical perspectives and arguments, we propose that the emergent typology of ‘relational welfare’ should be addressed and further developed through innovative practices, policies and empirical research. We especially encourage developmental and research strategies with a transformative purpose, radically attending to human relationships aligned with a participatory approach. Equity in health and wellbeing is thus framed as something more than social security for those left behind. Shifting the discourse in public health from a ‘health problem’ to a ‘village problem’ [104] has the potential to nurture social transformation towards securing participatory parity and development of capabilities to achieve wellbeing. Here, equity in health and wellbeing is instead (re-)framed as vital to securing sustainable development, community resilience and trust, and shared relational and societal responsibility. A movement towards universal and relational welfare has the potential to place fair societies and healthy lives at the heart of public value co-creation and become a viable framework for future research and practice. By outlining a typology and framework for developing relational welfare that builds on current developments in public health and the public sector, we hope to stimulate dialogue, new thought and innovation aimed at promoting social justice, health and wellbeing for all.
